# A population-based analysis of outcomes for small cell carcinoma of the breast by tumor stage and the use of radiation therapy

**DOI:** 10.1186/s40064-015-0913-y

**Published:** 2015-03-21

**Authors:** Felicia Hare, Smith Giri, Jashmin K Patel, Andrew Hahn, Michael G Martin

**Affiliations:** Department of Internal Medicine, The University of Tennessee Health Science Center, Memphis, USA; Department of Hematology/Oncology, The West Cancer Center/University of Tennessee Health Science Center, 1588 Union Ave., Memphis, TN 38104 USA

**Keywords:** Small cell carcinoma, Breast, Neuroendocrine, Radiation, Prognosis

## Abstract

**Purpose:**

Primary small cell carcinomas of the breast (SCCB) are rare tumors with limited data on outcomes and treatment strategies. Using a population based approach, we aimed to study outcomes of SCCB and determine whether the use of radiation therapy is associated with better survival among patients with SCCB.

**Methods:**

Using the Surveillance, Epidemiology and End Results (SEER) registry, we identified patients with SCCB between1973 and 2010. We examined the stage specific survival of these patients and compared it to the stage specific survival of small cell lung cancer (SCLC) from the SEER database over the same accrual period. We further analyzed the impact of radiation therapy on overall survival for SCCB patients using a univariate and multivariate approach.

**Results:**

A total of 199 patients with primary SCCB with staging were identified during the study period. Eighty-four patients (42%) had localized disease, 77 (39%) had regional disease and 38 (19%) had distant disease. For comparison, 81,933 patients with SCLC were identified. Outcomes were superior for patients with SCCB with localized (150 vs. 16 months, p < 0.01) and regional disease (56 vs. 13 months, p < 0.01), but not distant disease (7 vs. 7 months, p = 0.43). Use of radiation therapy was not associated with a significant difference in OS for patients with either localized (202 vs. 147 months, p = 0.48) or regional (52 vs. 75 months, p = 0.650) disease.

**Conclusions:**

SCCB has a more favorable prognosis by stage for localized and regional disease than SCLC. Adjuvant radiation is not associated with an improvement in survival for patients with localized or regional SCCB in this dataset.

## Background

Small cell carcinomas (SCC) are poorly differentiated neuroendocrine tumors that arise predominantly in the lungs (Grossman et al. 2011). Extrapulmonary small cell carcinomas (EPSCC) comprise ~2.5-5% of all SCC (van der Heijden and Heijdra 2005). While EPSCCs may occur at various sites, small cell cancer of the breast (SCCB) makes up ~4-10% of all EPSCC (Grossman et al. 2011). Overall, neuroendocrine breast cancers comprise about 2-5% of all breast cancer cases (Boyd and Hayes 2012).

Due to the rarity of SCCB, outcomes and treatment protocols are largely undefined. Treatment may include surgery and adjuvant chemotherapy/radiation therapy depending on tumor size and lymph node status; hormonal therapy is added if the tumor expresses the appropriate receptors (Adams et al. 2014; Adegbola et al. 2005; Shin et al. 2000). Adjuvant chemotherapy regimens include a platinum agent and etoposide since biologic markers of SCCB are similar to that of small cell cancer of the lung (SCLC) (Abbasi et al. 2013; Adegbola et al. 2005; Ge et al. 2012; Ochoa et al. 2012; Sanguinetti et al. 2013; Suhani et al. 2014). The role of radiation therapy in the treatment of SCCB remains controversial; there are no controlled trials definitively highlighting its benefit and or its effect on median overall survival (OS).

Using a population-based approach in the US, we aimed to study the overall and stage specific outcomes of patients with SCCB and identify the role of radiation therapy in the management of these cases.

## Methods

We utilized the Surveillance, Epidemiology and End Results (SEER) 18 database to identify all patients with primary SCCB between 1973–2010 (Surveillance and End Results (SEER) Program 2013). The SEER database includes data from nine population-based registries covering 1990–1999 and 18 covering 2000–2009, which covers approximately 26% of cancer patients in the US. It classifies cancer histology and topography information on the basis of the third edition of the International Classifications of Diseases for Oncology (ICD-O-3).

Cases of SCCB were identified in the SEER database using the appropriate ICD-O-3 codes for small cell cancer and oat cell cancer, which were 8041/3 and 8043/3. The results were further categorized by primary site breast using the codes C500-C506 and C508-C509. Cases with more than one primary were excluded. Patients identified as having SCCB were further classified as having localized, regional, and distant disease per SEER staging, which does not list staging in traditional clinical stages I-IV.

For comparison purposes, all cases of primary small cell lung cancer (SCLC) were identified during the same study period. These cases were identified using location codes C340-C343 and C348 respectively. SCLC patients were further classified as having localized, regional, and distant disease based on SEER summary stage.

Statistical analyses were conducted using Graph Pad Prism 6 and Statistical Package for Social Sciences (SPSS) version 21.0 (IBM Corporation, Armonk NY). Correlations between categorical variables were made using the chi-squared test. Median survivals were calculated using the Kaplan-Meier method. Differences in survival were computed using log-rank test (Mantel-Cox). Cox regression analysis was used for multivariate analysis using age, gender, race, tumor stage and the use of surgery and radiation therapy. All p-values were 2-sided and the level of significance was at 0.05.

## Results

A total of 199 patients with primary SCCB were identified using the study criteria. The median age was 65 years (range 28–97) and 98% were females. Among these patients, 84 (42%) had localized disease, 77 (39%) had regional disease and 38 (19%) had distant disease. Breast surgery was undertaken in 95% of patients with localized disease and 88% of patients with regional disease. A total of 69 patients (35.9%) received radiation therapy. A comparison of various demographic and clinical characteristics among patients with and without radiation therapy is shown in Table 1.

**Table 1:** **Comparison of demographic and clinical characteristics among patients treated with and without radiation therapy**

**Category**	**No Radiation therapy**	**Radiation therapy**	***P*** **value**
Number of patients*	123	69	
Median age at diagnosis, yr	68 (37–97)	61 (28–88)	0.02
Median survival, m	58	69	0.70
Race			0.52
- White	109 (88.6%)	59 (85.5%)	
- Black	10 (8.1%)	10 (14.5%)	
- Others	3 (2.4%)	0	
- Unknown	1 (0.8%)	0	
Female %	119 (96.7%)	69 (100%)	0.28
Surgery			0.90
- Yes	99 (80.5)	53 (76.8%)	
- No	21 (17.1%)	15 (21.7%)	
- Unknown	3 (2.4%)	1 (1.5%)	
Staging			0.62
- Distant	26 (21.1%)	11 (15.9%)	
- Localized	54 (43.9%)	28 (40.6%)	
- Regional	43 (35%)	30 (43.5%)	

yr, year; m, months.

* the use of radiation therapy was unknown in 7 patients.

**Figure 1:**
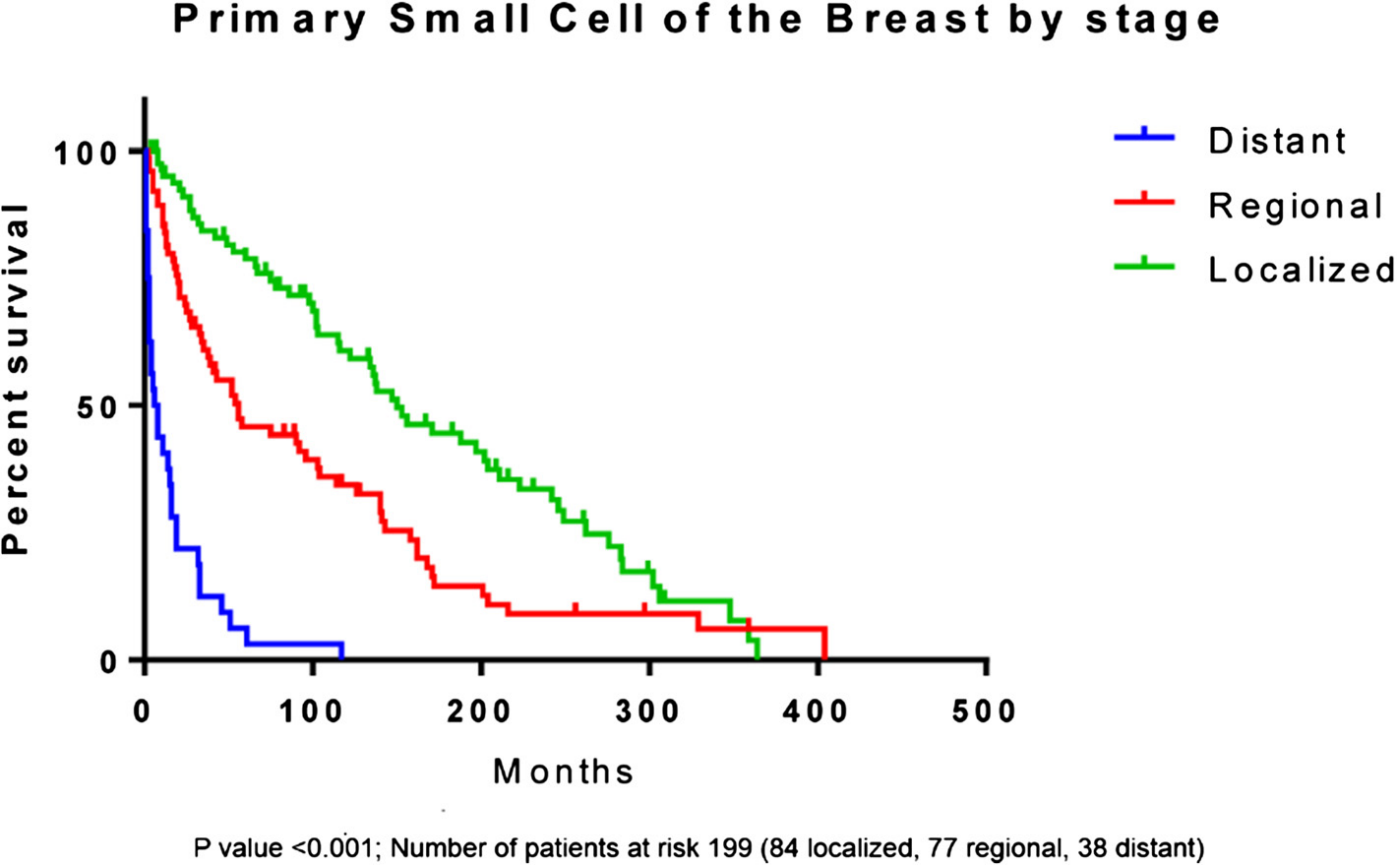
**Kaplan Meier Survival curves of primary small cell cancer of the breast by stage.** Log rank test was statistically significant with p value <0.001.

**Figure 2:**
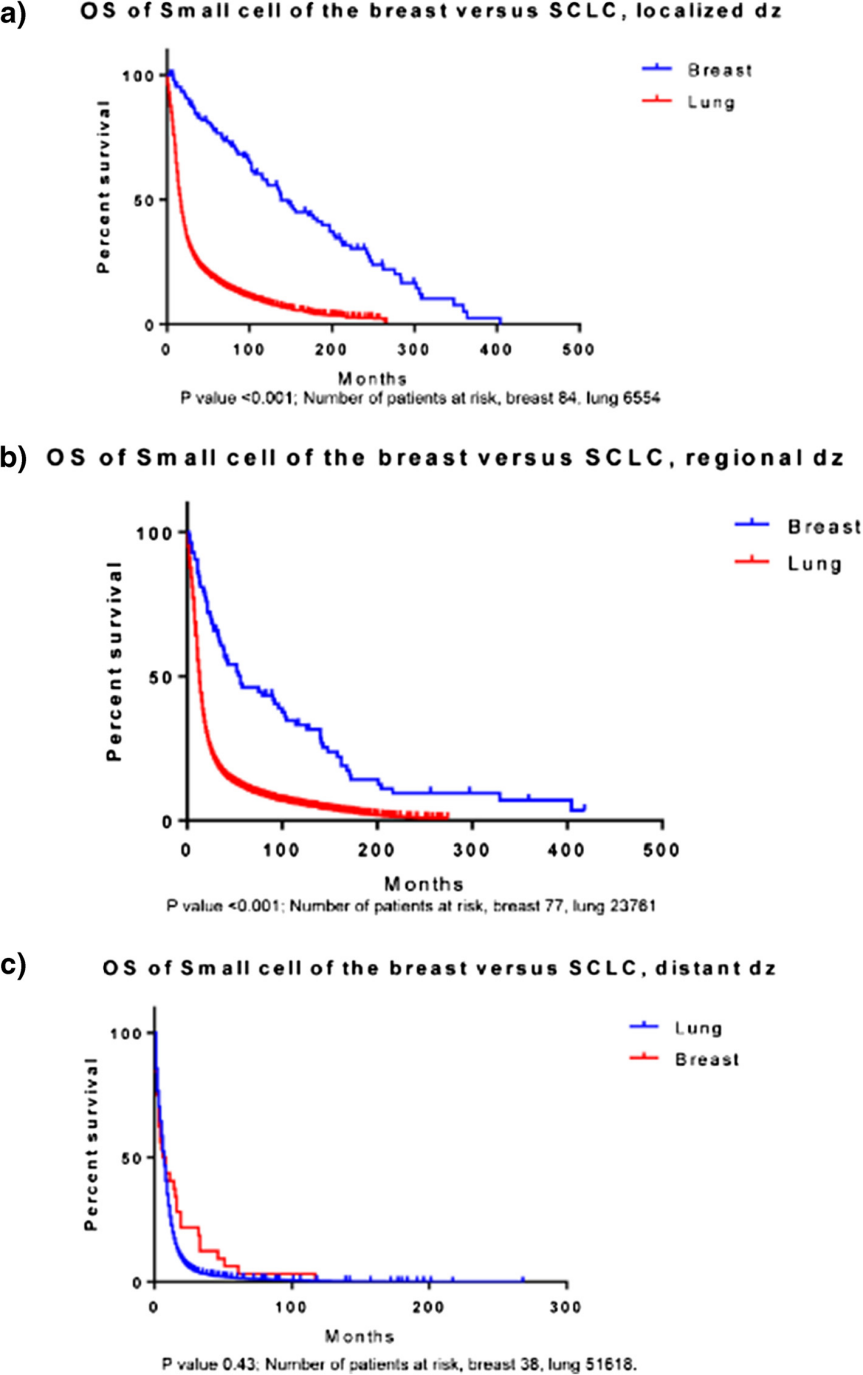
**Kaplan Meier Survival curves of primary small cell cancer of the breast and small cell cancer of the lung according to different stages. a:** Localized disease **b:** Regional disease. **c:** Distant disease. The survival curves were significantly different for localized and regional disease (p value <0.001 in both cases) but not for distant disease (p value 0.43).

The median overall survival (OS) varied by stage and was found to be 150 months (m), 56 m and 7 m (p < 0.001) for localized, regional, and distant disease respectively (Figure 1). A total of 81,933 cases of SCLC were identified during the same time period for comparison. The median OS was higher for patients with SCCB as compared to SCLC in patients with localized (150 m vs. 16 m, p < 0.001) (Figure 2a) and regional disease (56 m vs. 13 m, p < 0.001) (Figure 2b). However, the median OS for distant disease was similar for both SCCB and SCLC (7 m vs. 7 m, p = 0.43) (Figure 2c).

**Figure 3:**
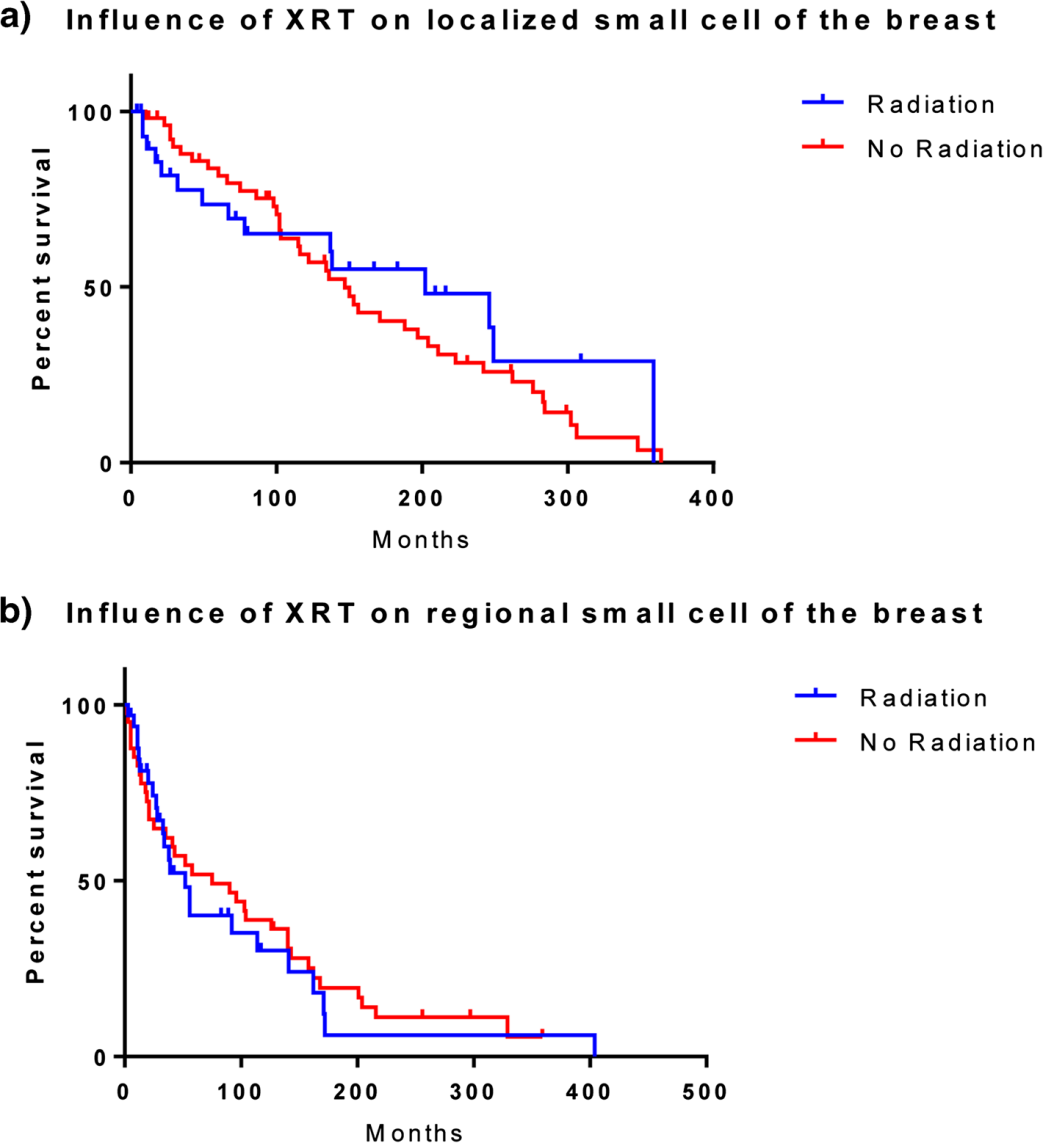
**Kaplan Meier Survival curves of primary small cell cancer of the breast with and without radiation. a:** Localized disease **b:** Regional disease.

In univariate analysis, no significant difference between the median OS of patients treated with and without radiation therapy was noted. On subgroup analysis, radiation therapy was not associated with significantly different median OS for patients with either localized (202 m vs. 147 m, p = 0.477) (Figure 3a) or regional disease (52 m vs. 75 m, p = 0.650) (Figure 3b). The difference in survival remained non-significant after adjusting for age, race and receipt of surgery in the multivariate analysis (Tables 2 and 3). Further, we tested if the combination of radiation therapy with surgery led to any survival differences in patients with local and regional disease by testing the interactions of these two variables in the multivariate model. However, the differences in OS continued to remain non-significant.

**Table 2:** **Multivariate Cox proportional hazard regression model for patients with localized SCCB**

**Category** ^*^	**HR**	**95% CI of HR**	***P*** **value**
Radiation therapy vs no radiation	0.68	0.36-1.31	0.25
Age group	-	-	0.01
- (50–70) vs <50 years	0.88	0.29-2.59	
- > 70 years vs <50 years	2.34	0.82-6.98	
Race	-	-	0.27
- Black versus white	2.15	0.27-16.84	0.46
- Others versus white	0.08	0.01-2.42	0.15
Surgery vs no surgery	0.01	0.01-0.25	<0.01

HR, hazard ratio; CI, confidence interval; vs, versus.

* a total of 81 cases included in this multivariate analysis.

**Table 3:** **Multivariate Cox proportional hazard regression model for patients with regional SCCB**

Category*	**HR**	**95% CI of HR**	***P*** **value**
Radiation therapy vs no radiation	0.97	0.55-1.71	0.93
Age group	-	-	0.01
- (50–70) vs <50 years	2.45	1.10-5.46	0.03
- > 70 vs <50 years	3.24	1.46-7.17	<0.01
Race	-	-	<0.01
- Black versus white -	4.59	1.75-4.59	<0.01
Others versus white	0.40	0.05-3.10	0.03
Surgery vs no surgery	0.72	0.29-1.74	0.47

HR, hazard ratio; CI, confidence interval; vs, versus.

* a total of 72 cases included in this multivariate analysis.

## Discussion

Primary SCCB is a rare disease with limited available data. Many of the reports in the literature are case reports or small case series and much of the information is anecdotal. The paucity of data creates difficulties in prognostication and treatment strategies, leaving outcomes largely undefined. Our study provides limited insight into the management of this rare disease by revealing the outcomes of SCCB by stage and showing no correlation between treatment with radiation therapy and overall survival, especially in patients with earlier disease.

Studies have shown that SCCB bears a clinical resemblance to more common forms of breast cancer, while also sharing histologic and morphologic features with SCLC (Adegbola et al. 2005; Ge et al. 2012; Jochems and Tjalma 2004). While some sources show that SCCB is as aggressive as its pulmonary counterpart, others have suggested SCCB has a more favorable prognosis, especially if caught in the early stages of the disease (Adams et al. 2014; Adegbola et al. 2005; Shin et al. 2000; Rovera et al. 2008; Jochems and Tjalma 2004; Yerushalmi et al. 2009). In contrast to SCLC, our results show that SCCB carries a more favorable prognosis in localized and regional disease. However, distant SCCB has dismal outcomes similar to SCLC. In an earlier study using the SEER registry, Grossman et al. showed that SCCB has the highest overall 5- and 10- year survival among all cases of EPSCC (Grossman et al. 2011). Another population-based study from the UK showed similar results (Wong et al. 2009). Studies have shown that EPSCC cases tend to present at earlier stages as compared to patients with SCLC (Subramanian et al. 2008). This translates into a better overall prognosis in patients with SCCB as compared to other forms of small cell cancer. Timely detection seems to be the key as the median overall survival drastically declines as the cancer metastasizes.

Since SCCB is rare and has not been extensively studied, treatment strategies are numerous and no recommended treatment protocol exists to guide clinicians. A combination of surgery, ranging from breast conserving to modified radical mastectomy, with adjuvant chemotherapy has been suggested in the treatment of localized and regional SCCB (Adams et al. 2014; Adegbola et al. 2005; Shin et al. 2000; Ge et al. 2012; Zekioglu et al. 2003; Murthy et al. 2013; Yildirim et al. 2011; Lopez-Bonet et al. 2008; Nicoletti et al. 2010; Haji et al. 2009; Navrozoglou et al. 2011; Yamasaki et al. 2000; Wei et al. 2010). The use of radiation therapy remains controversial in the literature (Wei et al. 2010; Yildirim et al. 2011; Navrozoglou et al. 2011). In our study, no association existed between overall survival for SCCB and treatment with radiation therapy, especially among patients with localized or regional disease. A prior study by Grossman et al. had shown a beneficial role for surgery and radiation therapy among all cases of EPSCC. However, the same study failed to show a significant survival benefit among the subgroup of patients with SCCB, which is in agreement with our findings (Abbasi et al. 2013).

It should be noted that our data for radiation therapy in SCCB is from a non-randomized population based sample. Despite performing a multivariate analysis for several risk factors affecting survival, we acknowledge our limitation of not including other variables, such as tumor size, tumor grade, chemotherapy usage, and hormone receptor status, which may have affected our observations. Other limitations of this study include the inability to verify the accuracy of coding; however, SEER databases are rigorously maintained and undergo quality monitoring. The strengths of our study include a large sample size in a population-based setting that enables us to study this rare malignancy.

## Conclusions

In summary, primary SCCB is an extremely rare form of breast carcinoma, which presents challenges for diagnosis, prediction of outcomes, and overall treatment strategies. Based on our SEER database review, SCCB has a more favorable prognosis than SCLC for localized and regional malignancy. The reasons for this cannot be elucidated at this time without further study of this rare carcinoma. However, it highlights the importance of early screening measures in overall survival because once the disease has progressed to a distant site, the prognosis is no longer appreciably improved over that of SCLC. The lack of a well-defined treatment protocol poses a difficulty for clinicians. This population-based database review shows no association between the use of radiation therapy for localized or regional SCCB and overall survival, which brings into question the use of radiation in treatment protocols.
